# GDF-15 and hepcidin as a therapeutic target for anemia in chronic kidney disease

**DOI:** 10.1186/s13052-023-01505-9

**Published:** 2023-08-30

**Authors:** Naglaa Makram Farag, Mahmoud Mousa, Eman Elsayed, Ahlam Ismeil

**Affiliations:** 1 Clinical Pathology, Minia University, Minia, Egypt; 2 Pediatrics Dep., Minia University, Minia, Egypt

**Keywords:** Chronic kidney disease, GDF-15, Hepcidin, Anaemia

## Abstract

**Background:**

Anaemia is a common presenting feature among patients with chronic kidney disease (CKD) and associated with poor clinical outcomes. We evaluated the diagnostic validity of growth differentiation factor-15 (GDF-15) and hepcidin as it is not clear if they are useful as a biomarkers of anaemia among non-dialysis CKD egyptian patients.

**Method:**

An analytical cross-sectional study was conducted among non-dialysis CKD patients (*n* = 60) and apparently healthy controls (*n* = 28) at Minia University maternity & children Hospital. Serum levels of GDF-15 and hepcidin were determined. Predictive logistic regression models were built and post estimation receiver operator characteristics were determined to evaluate diagnostic validity of hepcidin and GDF-15 for iron deficiency anaemia.

**Results:**

Hepcidin and GDF-15 are significantly higher in cases than control p value (0.047 < 0.0001) respectively. The predictive value of diagnosing anaemia among CKD patients using hepcidin and GDF-15 was 72.0%, 70.0%. There was a weak negative correlation between hepcidin levels and glomerular filtration rate GFR (*r* = -.175, *p* = 0.105) in CKD patients, and significant correlation between serum GDF-15 and haemoglobin (*r* = -0.897, *p* < 0.0001), ferritin (*r* = 0.489, *P* < 0.000), Iron (*r* = -0.314, *P* = 0.002), CRP (*r* = 0.409, *P* < 0.0001).

**Conclusion:**

Hepcidin and GDF-15 is a potential biomarker for predicting anaemia connected with inflammation among CKD Egyptian patients.

## Background

CKD patients are at risk of developing anemia which associated with a reduced quality of life and a worse survival [1]. Anemia prevalenc e in CKD is twice as in general population and more severe as e GFR declines [2]. The prevalence of anemia raised with the progression of CKD: 8.4% at stage 1 to 53.4% at stage 5 [3].

Anemia of chronic disease as well as iron deficiency are conditions that differentiating between them is quite complicated [4]. At the population level and in clinical practice, Haemglobin concentration is the most common indicator and assessment method used to define anemia**.** Although indices exist in clinical practice for distinguishing anemia etiology but their reliability for discriminating between causes varies [5]. Also, ferritin and Transferrin saturation (TSAT) both have limitations as indicators of iron stores. Ferritin is difficult to interpret as an independent marker of iron status as it is an acute-phase reactant [6]. Production of transferrin is also affected by inflammation, although, in contrast to ferritin, inflammation decreases transferrin production, resulting in misleadingly elevated TSAT values [7].

Iron deficiency is particularly common in patients with chronic diseases accompanied by inflammation, such as chronic kidney disease. The association between iron and inflammation remained unclear until the discovery of hepcidin which appears to be an emerging biomarker for anemia in CKD. It is an acute phase protein and one regulator of iron metabolism, It inhibits iron transport by binding to the iron export channel ferroportin resulting in ferroportin breakdown in lysosomes. Inhibiting ferroportin prevents iron from being exported from the cell [8]. Hepcidin synthesis and secretion by the liver is controlled by iron stores within macrophages, inflammation, hypoxia, and erythropoiesis [9]. Several studies have shown the correlation between hepcidin and ferritin. However, hepcidin is more superior in describing iron status. Therefore, hepcidin is a potential marker for iron status [10].

Severe anemia is associated with low hepcidin levels, even in the presence of inflammation Erythroferrone, produced in erythroblasts, has been identified as inhibiting hepcidin and so providing more iron for hemoglobin synthesis in situations such as stress erythropoiesis [11]. Also, acute inflammation contributes to anemia severity as production of cytokines increases hepcidin level, and may activate erythrophagocytosis [2]. Increasing hepcidin levels may not only reduce iron overload but also partially control ineffective erythropoiesis in iron loading anemias [11].

GDF-15, an anti-inflammatory cytokine, is a significant regulator of hepcidin and a strong positive correlation has been reported between them in anemic patients [12, 13]. In addition, Erythroblasts secrete GDF-15, which in turn suppresses hepcidin expression and decreases iron stores [14]. The role of GDF-15 in the evolution of anemia is still controversial. However, studies demonstrating a relationship between GDF-15 expression and serum iron parameters will assist to shed more light on the investigation, treatment and monitoring of anemia in CKD patients [15]. The aim of this study was to assess their correlation with inflammatory parameters and biomarkers of iron metabolism in CKD as the previous studies concerned only patients with end stage kidney disease or patients on renal replacement therapy.

### Subjects and methods

This study was carried out in Pediatric department in Minia University maternity & children hospital from Augest 2021 to February 2022, included 60 CKD patients with anemia and 28 apparently healthy children matching age and sex. The studied cases were subjected to General and systemic examinations, Glomerular filtration rate (GFR) was determined by the Chronic Kidney Disease Epidemiology Collaboration (CKD-EPI) equation for Egfr.

### Inclusion criteria

Chronic renal failure on dialysis, chronic forms of nephrotic syndrome and glomerulonephritis cause CKD, renal congenital anomalies as obstructive nephropathy, renal hypoplasia and neurogenic bladder, Chronic urinary tract infections causes CKD. The children were in a stable phase of their CKD.

### Exclusion criteria

Active infection, cancer, acute cardiovascular complications (uncontrolled hypertension acute coronary syndrome, and acute heart failure).

### Sample collection

7 mL venous blood samples were withdrawn by venipuncture, divided as follows; 2 ml specimens were collected into the K2 EDTA tube for Complete Blood Count (CBC), 5 mL specimens were collected into plain chemistry tube to clot and sera were separated without delay (immediate centrifugation at 3000 RPM; 15 min), then divided into 3 portions; one for CRP, urea and creatinine, one for iron profile (serum iron, total iron binding capacity, serum ferritin) and the last portion aliquoted and stored at -80 °C until analysis for the assessment of hepcidin and GDF 15.

CBC was measured using Sysmex diagnostic, USA. We determined chemistry tests, iron, total iron-binding capacity (TIBC) using SAL 6000 modular system fully automated machine according to the manufacturer’s instructions (reference range 100 to 120 µg/dL) and TIBC assay was (reference range 250 to 400 µg/dL) to evaluate the iron status and its binding capacity in the serum. Serum ferritin was measured by Elecsys ferritin assay using chemiluminescence immunoassay based on sandwich principle in electro Cobas e 411 fully automated Electro Chemiluminiscence Analyser (reference range 25–250 µg/L). TSAT was calculated as the ratio of serum iron and TIBC and expressed as a percentage. CRP Elisa Kit- LDN Labor Diagnostika Nord GmbH&Co KG, Nordhorn, Germany) was used.

Serum levels of Hepcidin by (EIA Kit, Peninsula Laboratories, LLC, Bachem Group, USA) (reference range 13.3 to 54.4 ng/mL) and GDF-15 (Bioassay Technology Laboratory, Yangpu, Shanghai, China) A Solid Phase Sandwich ELISA has been shown to accurately quantitate them with intra- and inter-assay CVs of < 10% and < 12%, respectively. The sensitivity down to 5.57 ng/L with an assay range: 10—3000 ng/LGDF-15 levels in healthy individuals and patients varied widely across studies, partly due to the use of different assays, and might therefore not be comparable between studies.

### Statistical analysis

The analysis of the data was carried out using IBM SPSS version 26.0 statistical package software (IBM; Armonk, New York, USA(. Normality of the data was tested using Kolmogorov–Smirnov test. Data were expressed as median (IQR) for non-parametric quantitative data, in addition to both number and percentage for qualitative data. Mann Whitney test was done for non-parametric quantitative data between the two groups. The Chi square test or Fisher’s exact test were used to compare categorical variables. Spearman’s rank correlation was done for non-parametric data. Receiver operating characteristic (ROC) curve analysis was done. A p-value less than 0.05 was considered significant.

## Results

Table 1 showed that demographic and clinical data of patients and control had significant difference regarding age, weight and height [0.049, 0.02*, 0.003*] respectively. Also, median levels of serum GDF-15 among patients were higher as compared to controls. Table 2 showed significant difference in all lab parameters [*p* value < 0.001] except ALT& AST and transferrin. Further sub-analysis showed that median serum hepcidin and GDF-15 level is higher and statistically significant among participants with higher ferritin level Table 3**.** For every ng/ml increase in serum ferritin level among CKD patients, log hepcidin increased by 0.00251 (β = 0.00251, *P*-value < 0.0001), In other words, hepcidin levels increased by 100 raised to 0.00389 units for every ng/ml increase in serum ferritin. Log GDF-15 decreased with every unit increase of MCHC (β = 0.0196, *P*-value = 0.005) Table 4.

**Table 1:** Demographic and clinical data of patients and control

		**Group I (Cases)** ***N* = 60**	**Group II (Control)** ***N* = 28**	***p* value**
Age (years)	Median(IQR)	10.0 (8.0 – 13.0)	12.0 (10.3 – 13.0)	0.049*
Males		36 (60.0%)	11 (39.3%)	0.07
Females	N%	24 (40.0%)	17 (60.7%)	
Rural		46 (76.7%)	19 (67.9%)	0.381
Urban	N%	14 (23.3%)	9 (32.1%)	
Weight (kg)	Median(IQR)	29.0 (23.0 – 35.5)	33.0 (30.0 – 35.8)	0.02*
Height (cm)	Median(IQR)	136.0 (122.3 – 144.0)	144.0 (135.8 – 148.0)	0.003*
BMI (kg/m2)	Median(IQR)	16.0 (14.5 – 18.4)	15.8 (15.2 – 17.5)	0.964

Mann–Whitney test for non-parametric quantitative data between the two groups *: Significant level at P value < *0.05*

**Table 2:** Laboratory data in patients and control group

Median (IQR)	Group I (Cases *N* = 60	Group II (Control) *N* = 28	*p* value
**Hb (g/dL)**	9.8 (9.0—10.5)	12.1 (11.9 – 12.3)	< 0.0001*
**MCV** (fL)	85.0 (79.3 – 88.0)	88.0 (85.0 – 92.0)	0.003*
**MCH**	27.0 (26.0 – 29.0)	28.5 (28.0 – 30.8)	< 0.001*
**MCHC**	32.0 (30.3 – 33.0)	33.0 (33.0 – 35.0)	< 0.0001*
**Urea** (mg/dl)	96.5 (39.8 – 124.3)	25.5 (25.0 – 28.0)	< 0.0001*
**Creat** (mg/dl)	2.2 (0.9 – 4.3)	0.6 (0.5 – 0.7)	< 0.0001*
**eGFR (ml/min)**	28.6 (22.3 – 67.2)	130.0 (120.8 – 152.3)	< 0.0001*
**A/C ratio**	322.0 (153.0 – 1073.0)	–––	**–––-**
**ALT (U/L)**	22.0 (15.0 – 30.0)	22.0 (18.5 – 27.0)	0.689
**AST (U/L)**	25.0 (21.0 – 33.0)	23.5 (18.8 – 27.8)	0.119
**Iron (µg/dL)**	52.0 (36.0 – 88.3)	100.0 (85.8 – 106.8)	< 0.0001*
**Ferritin (μg/l)**	755.0 (181.5 – 1330.5)	95.0 (88.0 – 102.0)	< 0.0001*
**TIBC (µg/dL)**	254.0 (187.8 – 287.5)	161.5 (153.5 – 189.5)	< 0.0001*
**Transferrin**	25.0 (16.3 – 42.0)	25.0 (19.0 – 34.3)	0.74
**CRP** (mg/l)	12.0 (3 – 24.0)	3 (2—5)	< 0.0001*
**Hepcidin (ng/mL)**	338.5 (276.0 – 505.5)	303.0 (215.0 – 387.0)	0.047*
**GDF** (ng/L)	1277.2 (1072.3 – 1376.6)	401.6 (298.6 – 594.5)	< 0.0001*

* significant level at *P* value <0.05

**Table 3:** Laboratory data in patients according ferretin level

Median(IQR)	Ferritin < 300	Ferritin > 300	p value
Hepcidin	285.0 (101.0 – 388.5)	361.0 (260.0 – 604.0)	< 0.01*
GDF-15	973.1 (298.6 – 594.5)	1039.8 (1072.3 – 1376.6)	< 0.001*
Hemoglobin	9.8 (9.1 – 10.6)	11.8 (10.2 – 12.2)	< 0.0001*
Ferritin	102.0 (91.0 – 258.0)	798.0 (338.0 – 1311.5)	< 0.001*
Iron	45.0 (35.5 – 60.5)	103.0 (85.0 – 118.0)	< 0.0001*
MCV	73.0 (69.5 – 75.0)	88.0 (82.0 – 91.0)	0.009*
MCH	24.0 (21.5 – 26.4)	28.0 (27.0 – 30.0)	0.031*
MCHC	30.0 (27.0 – 33.0)	33.0 (32.0 – 35.0)	0.032*

* significant level at *P* value <0.05

**Table 4:** Multiple linear predictors of log Hepcidin and logGDF-15 among CKD patients

	**Log hepcidin**		**Log GDF-15**	
	Coefficient	*P*-value	Coefficient	*P*-value
**Ferretin**	0.00251	< 0.0001	-0.00031	0.273
**MCHC**	-0.0276	0.303	0.0196	0.005
**MCV**	0.0075	0.617	-0.018	0.047

**Fig. 1:**
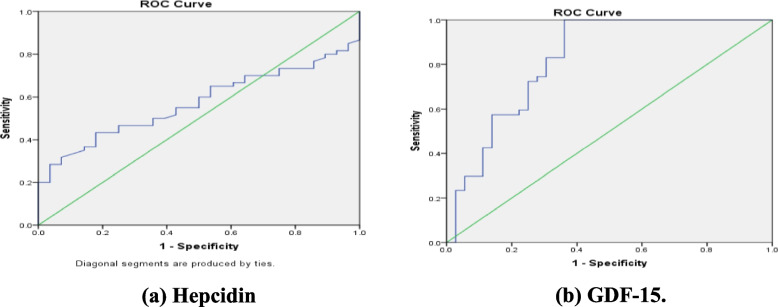
ROC curve Displaying the diagnostic accuracy to predict anemias (**a**) Hepcidin (**b**) GDF-15

The predictive value of hepcidin and GDF-15 for diagnosing anemia among CKD participants was 72.0%, 76.47% respectively. Using the non-covariate analysis and Younden’s index, the optimum cut-off value of GDF-15 for diagnosing anemia among CKD participants was 723 ng/l (at a sensitivity of 82.98%and specificity of87.11%) Similarly, the optimum cut-off for hepcidin was 209 ng/dl (at a sensitivity of 80.85 and specificity of 85.11); A combination of the two parameters did not improve significantly the diagnostic value of either of the two tests (Table 5, Fig. 1).

**Table 5:** Hepcidin & GDF-15 and different parameters for predicting anaemia

	**Sensitivity**	**Specificity**	**PPV**	**NPV**	**Accuracy**	***P*** **value**
**Hepcidin**	80.85%	85.11%	72.00%	74.29%	72.73%	< .001
**GDF**	82.98%	87.11%	76.47%	75.00%	75.90%	< 0.001*
**Hepcidin + GDF**	83.1%	87.93%	75.2%	76.21%	77.32%	< 0.01
**Hemoglobin**	72.34%	65.85%	70.83%	67.50%	69.32%	< .0091
**Iron**	85.11%	82.93%	85.11%	82.93%	84.09%	< .0091
**Ferritin**	40.43%	24.39%	38.00%	26.32%	32.95%	< .001

* significant level at *P* value <0.05

**Fig. 2:**
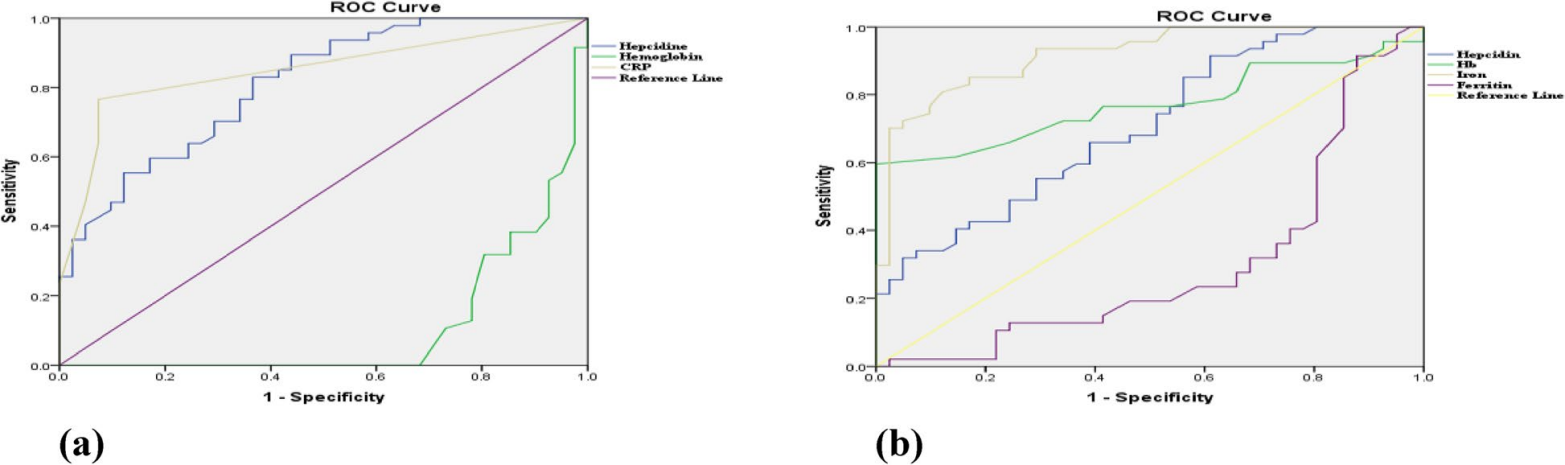
ROC curve of different parameters predicting types of anaemia

Receiver operating characteristic ROC curve displaying the diagnostic accuracy of hemoglobin, iron, ferritin and Hepcidin for predicting anemia in (**a**) cases with ferretin < 300 (**b**) cases with ferretin > 300 Fig. 2.

Factors found to be significantly associated with anemia by univariate analysis were entered the multivariable model to detect the independent risk factor for anaemia Table 6. Patients with low hemoglobin or hepcidin are more likely to have anemia (mostly iron defiency anemia) with p value of 0.008 for hemoglobin and < 0.001 for hepcidin while patients with high level ferritin and CRP are more likely to have anemia (anemia of chronic disease) with p value of < 0.0001 for ferritin and CRP. Patients with low level hepcidin and high level of GDF is more likely to have iron deficiency anemia with p value 0.007, 0.002 respectively. So, Hepcidin and GDF-15 were predicted by markers of inflammation: CRP&ferritin, Inflammation is correlated with iron status.

**Table 6:** Multivariate regression of variables associated with anaemia in CKD patients

**Variable**	**Univariate**			**Multivariate**		
	**Standardized coefficient** **Beta**	**t**	**p**	**Standardized coefficient** **Beta**	**t**	**p**
**GDF**	-0.35	-3.32	< 0.001*	-0.3	-3.2	0.002*
**Hepcidin**	-0.28	-2.73	0.008*	-0.26	-2.77	0.007*
**Hb**	-0.28	-2.73	0.008*	-0.34	-3.45	< 0.001*
**Iron**	0.16	1.52	0.133			
**Ferritin**	-0.4	-4.05	< 0.0001*	-0.19	-1.39	0.168
**CRP**	-0.39	-3.99	< 0.0001*	-0.19	-1.41	0.136

*Dependent variable* Hb

* significant level at *P* value <0.05

Hepcidin and GDF -15 were negatively correlated with GFR &HB and positively correlated with other markers shown in Table 7.

**Table 7:** Correlation of hepcidin and GDF -15 with other parameters

Correlation	Hepcidin		GDF	
	**r**	**p**	**r**	**p**
**Hepcidin**	---	---	0.03	0.785
**GDF**	0.03	0.785	---	---
**Hb**	-0.182	0.089	-0.897	< 0.0001*
**Iron**	0.334	< 0.001*	-0.314	0.002*
**Ferritin**	0.196	0.067	0.489	< 0.0001*
**Trans-ferritin**	0.092	0.395	0.085	0.461
**CRP**	0.218	0.042*	0.409	< 0.0001*
**GFR**	-0.175	0.105	-0.137	0.113

In CKD patients several significant correlations were observed as presented in the Table 7

* significant level at *P* value <0.05

## Discussion

To our knowledge, this is the first study to evaluate hepcidin and GDF-15 as markers of anemia among Egyptian with non-dialysis CKD. Serum hepcidin and GDF-15 predicted anemia among patients with CKD, with a predictive value of 72% and 76.47%, respectively. In addition, we showed a negative correlation between hepcidin and eGFR among CKD patients. Our observation agrees with findings by others [16, 17]. However, some authors found a positive correlation between hepcidin and GFR levels [18]. This suggests that the relationship between hepcidin and renal function is still unclear. Moreover, renal function plays a minimal role in hepcidin pathways which is largely dependent on ferritin metabolism. The sensitivity of the methods used may also play a role in the differences between our findings and others as we used ELISA, others used mass spectrometry [17, 19]. Our data supports published studies that reported higher levels of hepcidin in CKD [20]. In the setting of CKD, increase in serum hepcidin levels compared to controls might be due to inflammatory up regulation and decreased renal clearance of hepcidin which results in reduced availability of plasma iron and anemia [21]. We found that serum ferritin, the primary storage molecule for cellular iron, positively correlated with hepcidin in our CKD patients as previously documented [22]. The direct relationship of hepcidin with ferritin may represent a protective effect of hepcidin against iron overload [18]. However, there was correlation between hepcidin and inflammatory markers such as hsCRP this explain that CKD patients are prone to develop IDA due to the presence of persistent low-grade inflammation which induces Hepcidin and thereby mediates reticuloendothelial cell block.

Also, in our study serum GDF-15 are significantly elevated in CKD children than control and strongly correlated to CRP serum levels. There are possible mechanisms that may explain the findings of increased GDF-15 in anemia. First, GDF-15 may be an important mediator in a negative feedback pathway where it suppress high hepcidin levels in CKD patients with iron deficiency. Another explanation is that iron depletion could independently cause GDF-15 induction in the erythroid precursor cells as a result of iron sequestration in macrophages as GDF15 is a product of macrophages activated by pro-inflammatory cytokines that present in excess in CKD, Interestingly CRP induces GDF15 expression through the regulation of p53 binding sites in the GDF15 promoter and they are also relevant markers of inflammation [23].

Supporting the relationship of GDF-15 to the inflammatory status, We found a strong correlation between both serum ferritin and CRP with GDF-15. Moreover, median serum hepcidin and GDF-15 level is higher and statistically significant among participants with higher ferritin level > 300 μg/l. For every ng/ml increase in serum ferritin level among CKD patients, log hepcidin increased by 0.00251 (β = 0.00251, *P*-value < 0.0001), In other words, hepcidin levels increased by 100 raised to 0.00389 units for every ng/ml increase in serum ferritin. Log GDF-15 decreased with every unit increase of MCHC (β = 0.0196, *P*-value = 0.005) this explained by cytokine blocks hepcidin expression and increases iron absorption, thus leading to iron loading in these anemias [24]. Besides, in response to anemia, erythroblasts secretes GDF-15, which in turn suppresses hepcidin expression and decrease iron stores [24]. Yilmaz et al. and Wang et al. [23, 25], reported a significant correlation between GDF15 and serum ferritin but Li et al. [26], reported no significant correlation.

From our results, the high ferritin levels associated with CRP and GDF-15 may indicate the link between iron homeostasis and the inflammatory response through anemia of inflammation or chronic disease. Also, the diagnostic performance of GDF-15 as a marker of inflammation in CKD was comparable to CRP and better than ferritin that is mostly elevated by iron overload associating CKD, qualifying GDF-15 as a surrogate marker of inflammatory status in CKD. Similar results were found by Bargenda et al. [27], Thorsteinsdottir et al. [28]

Another important finding in this study is the negative correlation of GDF-15 with haemoglobin in CKD patients. This finding is consistent with others [26, 29], but is in disagreement with findings in non-CKD populations, where GDF-15 was positively correlated with haemoglobin [30, 31]. This difference could be explained by racial differences and diversity of ethnicity in the studied population.

We also found that GDF-15 predicted anemia at a cut-off value < 724 pg/ml with a predictive value of 70.0%, sensitivity of 82.98%, and specificity of 87.11%. In contrast, Tanno et al. [32]. found that there was no association between iron deficiency and serum GDF-15 levels. Our study, therefore, suggests that GDF-15 can be a useful diagnostic tool in patients with anemia. However, inflammation-mediated changes seen in iron homoeostasis may not induce the increased GDF-15 levels in patients with anemia, as serum ferritin levels did not correlate with GDF-15 levels in this study and as reported by Mast et al. [33]

Altough the major cause of anemia in this study was ACD, we still have to be aware of mixed anemia because it consists of one third of anemia in CKD. Treatment with erythropoietin stimulating agents (ESA) could increase Hb and ESA hypo or nonresponsive patients is commonly treated by increasing ESA dose. However, several clinical trials in adults have highlighted the side effects of ESA therapy, such as that affecting the cardiovascular and cerebrovascular system as well as death [34].

This study recommends further exploration for anemia in CKD, particularly in patients with no or less response to the standard therapy. The measurement of hepcidin and GDF-15 is highly recommended and can be standardized universally so that in future it could be clinically used routinely in those conditions.

## Conclusion

GDF-15 and hepcidin is involved in inflammatory response that play an important role in anemia. It could be a promising tools in predicting anemia in CKD patients. It could be a step in precision medicine and anti-cytokine treatment strategies in the future directions aiming at the treatment of inflammation-associated anemia and its subsequent complications. More extensive studies are necessary to determine a reliable cut-off value of serum hepcidin and GDF-15 in anemia diagnosis.

## Data Availability

Not applicable.

## Abbreviations

CKD: Chronic kidney disease
GDF-15: Growth differentiation factor-15
GFR: Glomerular filtration rate
TSAT: Transferrin saturation
